# Single-Cell Transcriptomics of Human Lymph Node Stroma

**DOI:** 10.1016/j.xpro.2020.100021

**Published:** 2020-06-03

**Authors:** Akira Takeda, Sirpa Jalkanen

**Affiliations:** 1 MediCity Research Laboratory and Institute of Biomedicine, University of Turku, Turku, Finland

## Abstract

Accumulating evidence indicates that the immune system is regulated not only by immune cells but also by stromal cells in the tissue microenvironment. Characterization of non-hematopoietic cells has not been performed in depth, since markers of the subsets are limited. Recent advances of single-cell technology allow researchers to characterize comprehensively the heterogeneity of stromal cells in an unbiased manner. In this article, we provide step-by-step protocols for cell preparation for single-cell RNA sequencing to characterize the heterogeneity of stroma in human lymph nodes. For complete details on the use and execution of this protocol, please refer to Takeda et al. (2019).

## Highlights

• Protocol to prepare human stromal and endothelial cells for single-cell RNA sequencing
• Gentle cell dissociation of human lymph nodes is critical for successful isolation
• Protocol can be used to characterize the heterogeneity of stroma in lymph nodes

## Graphical Abstract

**Figure:**
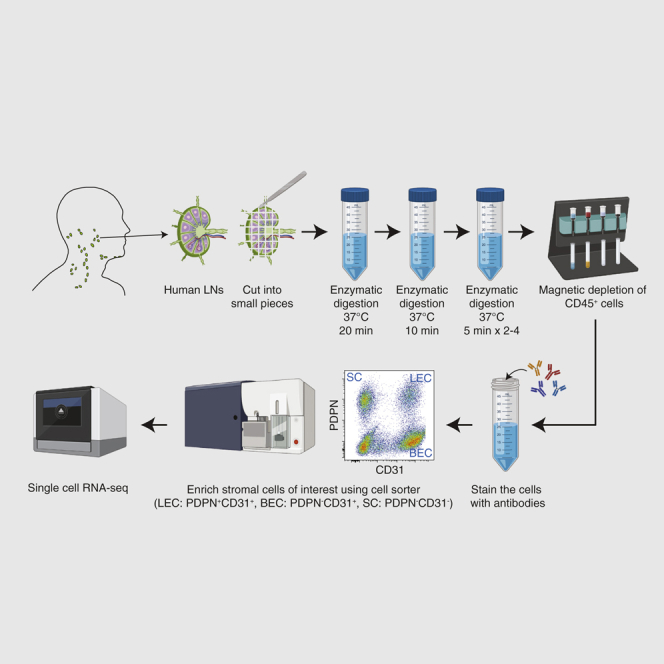

## Before You Begin

**TIMING: 1 h**

1. Prepare 15 ml enzyme mix consisted of RPMI, 0.8 mg/ml Dispase, 0.2 mg/ml collagenase P and 0.1 mg/ml DNase for each tissue.
2. Prepare PBS supplemented with 2% FCS and 2 mM EDTA.
3. Prepare phenol-red free DMEM supplemented with 2% FCS and 10 mM HEPES.
4. Set the temperature of the centrifuge to 4°C,
5. Set the temperature of a water bath to 37°C.
6. Coat a 1.5 ml tube for one sample with FCS by adding 1 ml of FCS and incubating for more than 12 hrs to prevent adhesion of sorted cells to tubes.

***Note:*** Proper isolation of human stromal cells is critical for generation of comprehensive single-cell data since insufficient tissue digestion lose certain cell subsets and excessive dissociation damage cells and affect cell viability. Currently available protocols regarding single-cell studies mainly address different single-cell RNA-seq (scRNA-seq) platforms, overall experimental design and data analysis and processing (Kulkarni et al., 2019, Lafzi et al., 2018, See et al., 2018). Here we mainly focus on cell isolation which is critical to comprehensively characterize stromal cell subsets.

## Key Resources Table

**Table:** 

REAGENT or RESOURCE	SOURCE	IDENTIFIER
**Antibodies**		

AF488-anti-human CD45	Biolegend	Cat# 304019; RRID: AB_493033
PE-anti-human podoplanin	Biolegend	Cat# 337004; RRID: AB_1595457
APC-anti-human CD31	Biolegend	Cat# 303115; RRID:AB_1877152

**Chemicals, Peptides, and Recombinant Proteins**		

RPMI	Sigma	Cat# R5886
Phenol-red free DMEM	Sigma	Cat# D1145
FCS	Sigma	Cat# F7524
HEPES	Lonza	Cat# BE17-737E
Dispase	Gibco	Cat# 17105-041
Collagenase P	Roche	Cat# 11213857001
DNase	Roche	Cat# 10104159001
DMEM	Sigma	Cat# D6429

**Critical Commercial Assays**		

EasySep human CD45 depletion kit	Stem Cell Technology	Cat# 17898
LIVE/DEAD Fixable Near-IR Dead Cell Stain kit	Thermo Fisher Scientific	Cat# L34975

**Other**		

100 μm cell strainer	BD	Cat# 352360
Water bath	N/A	N/A
Scalpels	Swann-Morton	Cat# 0503
Wide bore tips	Sartorius	Cat# 791021
Cell sorter	Sony	Cat# SH800S

## Materials and Equipment

Enzyme Mix- Make 15 ml for each tissue.

**Table:** 

RPMI
0.8 mg/ml Dispase
0.2 mg/ml collagenase-P
0.1 mg/ml DNase

Can be stored at 4 °C for up to 1 day.

## Step-by-Step Method Details

### Preparation of Single-Cell Suspension from Human Lymph Nodes

**TIMING: 2–3 h**

This step enzymatically dissociates cells from fresh human lymph nodes.

1. Immediately after surgery, transfer human lymph nodes (LNs) into 35 mm dishes filled with 5 ml RPMI on ice and cut into small pieces (around 0.5 mm^3^ cubes) using fine scalpels.
2. Add LN pieces into 5 ml of the enzyme mix in 15 ml tube and incubate at 37°C in a water bath for 20 min in total and gently invert the tube 2-3 times every 5 min.
3. After incubation, LN pieces are gently passed through a wide bore tip of a 1 ml pipette. Let fragments settle to the bottom for 30 sec, after which the enzyme mix was removed and added into the 15 ml tubes containing the cold PBS supplemented with 2% FCS and 2 mM EDTA (supernatant 1).
4. Add 5 ml of fresh enzyme mix into the tubes with LN fragments and incubate for 10 min at 37°C. Invert the tubes every 5 min.
5. After the incubation, mix the fragments strongly for 30 sec using a 1 ml pipette equipped with a wide bore tip. Let fragments settle down and remove the supernatant and add it to the new tube containing the cold PBS including 2% FCS and 2 mM EDTA (supernatant 2).
6. Add 5 ml of fresh enzyme mix to the tubes with LN fragments and incubate it at 37°C until the fragments were almost completely digested. Mix fragments vigorously every 5 min (usually 5 min x 2-4 times). Combine the dissociated single cell suspension with the supernatant 1 and 2, and centrifuge at 300 g, 4 °C and filter the cells with 100 μm cell strainer and centrifuge it.
7. Add phenol red-free DMEM supplemented with 2% FCS and 10 mM HEPES. Count the cell number. Typical viability at this point is 95%.

**CRITICAL:** Use phenol red-free DMEM containing 2% FCS and 10 mM HEPES instead of PBS containing FCS until cell sorting to keep the cell viability high.

### Deplete CD45^+^ Cells and Sort the Target Stromal Cells with a Cell-Sorter

**TIMING: 2–3 h**

This step enriches stromal cells via a depletion of hematopoietic cells.

**Figure 1:**
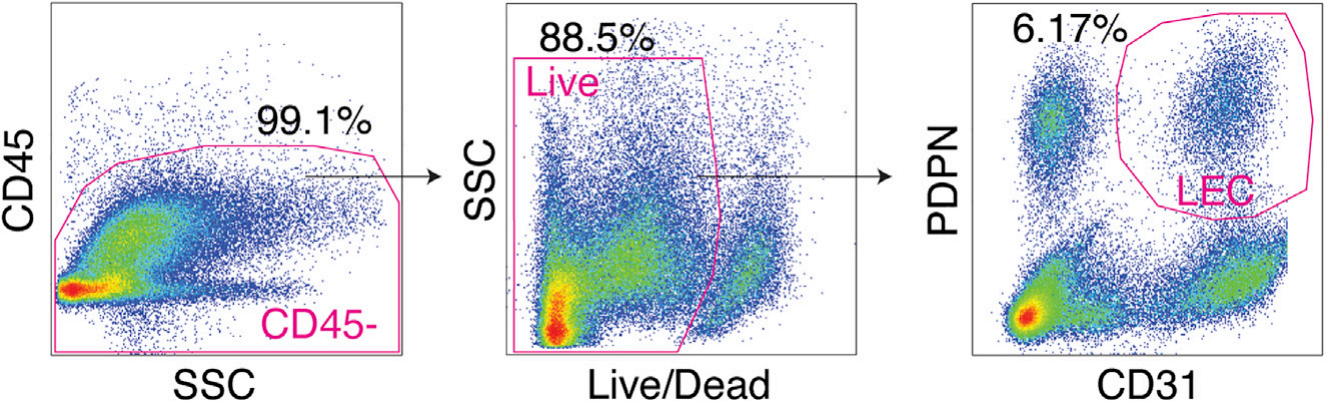
LEC Sorting from Human LNs for Single-Cell Analysis Gating strategy for isolation of LECs from human LNs. CD45^+^ cells and dead cells are excluded based on CD45 and Live/Dead dead cell stain, respectively. Live single CD45^-^PDPN^+^CD31^+^ are sorted for the single-cell analysis of human LECs. Adopt from Figure S1A of Takeda et al., 2019.

***Note:*** Viability differs between different subsets (Figures 2A and 2B). Stromal cells especially LECs (viability ~80%) are more prone to cell death than CD45^+^ leukocytes (viability ~95%). Even though live LECs are gated and sorted for scRNA-seq of LECs, viability of post-sort LECs is comparable to that of LECs after cell dissociation (Figure 2B). This indicates that FACS sorting damages stromal cells.

8. Deplete CD45^+^ cells with CD45^-^ negative cell isolation kit by following manufacture’s protocols. Use phenol red-free DMEM during isolation and incubate the cells at 4^°^C.
9. Incubate the CD45^-^ cells with fluorescence-conjugated antibodies including AF488-CD45, PE-podoplanin, APC-CD31 and LIVE/DEAD-Near IR for 30 min at 4^o^C.
10. Sort the cell-type of interest using a cell-sorter (e.g. SH800 from Sony) equipped with 100 μm chip. Sort PDPN^+^CD31^+^, PDPN^-^CD31^+^ or PDPN^+^CD31^-^ cells for isolation of lymphatic endothelial cells (LECs), blood endothelial cells (BECs) or non-endothelial stromal cells (SCs), respectively. Sort more than 20000 cells if possible into pre-coated 1.5 ml tubes containing 500 μl of DMEM supplemented with 10% FCS. Gate single cells with the FSC-H versus FCS-W plot and the SSC-H versus SSC-W plot to avoid doublets, and exclude dead cells based on LIVE-DEAD dead cell stain (Figure 1).

**Figure 2:**
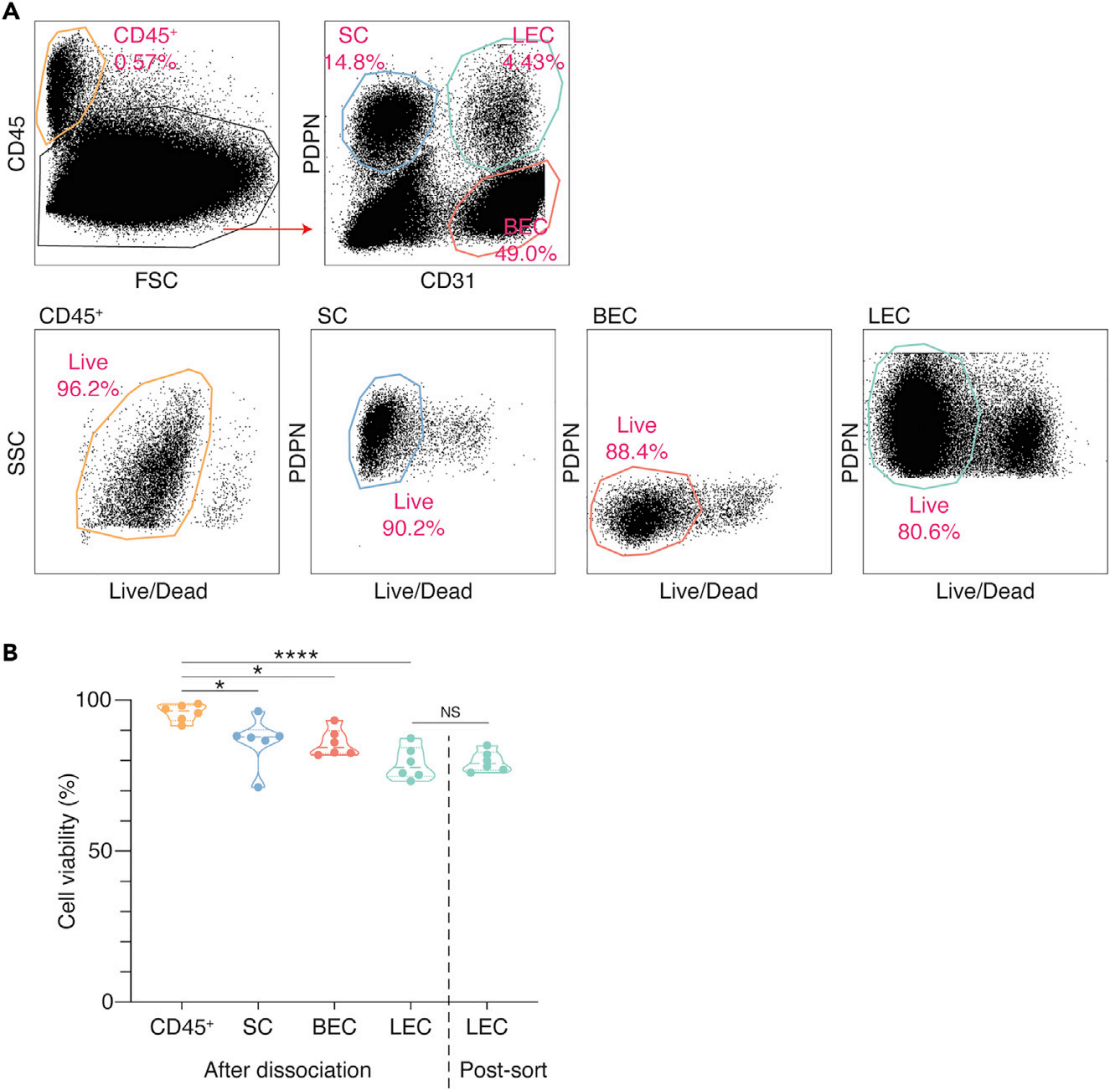
Viability of Dissociated Human LN Stromal Cell Subsets after Cell Dissociation and Cell Sorting (A and B) Viability of CD45^+^ cells, SCs, BECs and LECs dissociated from human LNs. (A) FACS plots. The images are representative of six biological replicates. (B) Comparison of viability of dissociated stromal cells and post-sort LECs. Viability of cells after dissociation were counted by FACS and viability of post-sort cells were counted with Trypan blue. The circles in the Violin plot indicate the number of biological replicates (n=6). ∗p < 0.05, ∗∗∗∗p < 0.0001.

**CRITICAL:** LIVE-DEAD dead cell stain-negative cells should be more than around 80 % of all the cells. Cell viability should be recorded pre and post-sorting (when counting cell number for library preparation) for proper metadata collection (Figure 2A). If the cell viability is low, final cell number will be low after quality check of scRNA-seq data and minor cell populations will not be detected.

### Library Preparation for Single-Cell RNA-Sequencing

Count cell number and prepare single-cell RNA-sequencing libraries.

11. Count sorted human stromal cells manually at least two times. Check the cell-viability with trypan blue and recode it as metadata.
12. Process the samples according to 10X Genomics User Guide.
13. Prepare single-cell RNA-sequencing libraries according to the manufacturer’s instruction from 10X Genomics (v2 or v3 chemistry).

## Expected Outcomes

Viability differs between different subsets in human LNs (Figures 2A and 2B). Stromal cells especially LECs are more prone to cell death than CD45^+^ leukocytes. Among LEC subtypes, there might be some more sensitive LEC subsets than others. Thus, frequency of subpopulations in scRNA-seq may not be equal to that in *in vivo* (Takeda et al., 2019). Even though live LECs are gated and sorted to enrich live LECs, viability of post-sort LECs is comparable to that of LECs after dissociation (Figure 2B). This may imply that FACS sorting damages subtypes of stromal cells. Thus, recording of viability as metadata is important and it should be taken into account when analyzing single-cell data.

## Limitations

Using 10X Genomics Chromium kit, we can analyze up to 10,000 cells with one reagent. If you want to comprehensively characterize non-hematopoietic cells in human LNs, you need to deplete hematopoietic cells since majority of cells (more than 90%) in LNs are lymphocytes. Subpopulations of stromal cells may be more prone to dissociation-associated cell death than other subsets. Thus, it is important to keep in mind that you might not detect rare and sensitive cell types depending on viability.

## Troubleshooting

### Problem

Single-cell RNA-seq is a powerful tool to investigate the cellular heterogeneity, developmental pathway and molecular interactions between cell subsets (Takeda et al., 2019, Velten et al., 2017, Vento-Tormo et al., 2018). However, interpretation of unbiased computational analysis is sometimes tricky. Cell stress caused by the enzymatic dissociation may induce transcriptome changes at certain populations and produce cell clusters that are unlikely to exist in the physiological conditions (van den Brink et al., 2017).

### Potential Solutions

It has been shown that dissociation of the tissue induces transcriptional changes in certain genes such as genes coding heat-shock proteins (van den Brink et al., 2017). Cell clusters highly expressing those genes can be removed from further analysis. Moreover, clusters that are not observed constantly between different samples may be removed. In this context, biological replicates (LNs from multiple patients) are crucial. To confirm the presence of heterogeneity detected by single-cell RNA-seq, other supporting information such as visualization of the subsets via immunostaining or in-situ hybridization is needed.

### Problem

The use of specific stromal cell markers may lead to loss of cell heterogeneity. It has been widely believed that LECs can be distinguished from BECs by expression of multiple molecules including LYVE1 and PROX1. However, the scRNA-seq of LECs sorted with anti-LYVE1 will show limited heterogeneity since LYVE1 is not expressed on all LEC subsets (Takeda et al., 2019).

### Potential Solutions

To avoid loss of heterogeneity by using selective markers, the use of general cell markers is recommended. For instance, PDPN and PROX1 are more general lymphatic markers than LYVE1. Our scRNA-seq of human LEC analysis also showed the difference of PDPN expression in six human LEC subsets. Thus, to comprehensively analyze the LEC population, PDPN low or intermediate cells should also be included for the analysis. Contaminated cells such as BECs and SCs can be removed in scRNA-seq analysis by use of BEC and SC markers (e.g. JAM2 and CCL19, respectively).
